# The Alphaviral Capsid Protein Inhibits IRAK1-Dependent TLR Signaling

**DOI:** 10.3390/v13030377

**Published:** 2021-02-27

**Authors:** V. Douglas Landers, Daniel W. Wilkey, Michael L. Merchant, Thomas C. Mitchell, Kevin J. Sokoloski

**Affiliations:** 1 Department of Microbiology and Immunology, School of Medicine, University of Louisville, Louisville, KY 40202, USA; v.landers@louisville.edu (V.D.L.); thomas.mitchell@louisville.edu (T.C.M.); 2 Center for Predictive Medicine and Emerging Infectious Diseases, University of Louisville, Louisville, KY 40202, USA; 3 Department of Medicine, Division of Nephrology and Hypertension, School of Medicine, University of Louisville, Louisville, KY 40202, USA; daniel.wilkey@louisville.edu (D.W.W.); michael.merchant@louisville.edu (M.L.M.)

**Keywords:** alphavirus, capsid, IRAK1, toll like receptors (TLR)

## Abstract

Alphaviruses are arthropod-borne RNA viruses which can cause either mild to severe febrile arthritis which may persist for months, or encephalitis which can lead to death or lifelong cognitive impairments. The non-assembly molecular role(s), functions, and protein–protein interactions of the alphavirus capsid proteins have been largely overlooked. Here we detail the use of a BioID2 biotin ligase system to identify the protein–protein interactions of the Sindbis virus capsid protein. These efforts led to the discovery of a series of novel host–pathogen interactions, including the identification of an interaction between the alphaviral capsid protein and the host IRAK1 protein. Importantly, this capsid–IRAK1 interaction is conserved across multiple alphavirus species, including arthritogenic alphaviruses SINV, Ross River virus, and Chikungunya virus; and encephalitic alphaviruses Eastern Equine Encephalitis virus, and Venezuelan Equine Encephalitis virus. The impact of the capsid–IRAK1 interaction was evaluated using a robust set of cellular model systems, leading to the realization that the alphaviral capsid protein specifically inhibits IRAK1-dependent signaling. This inhibition represents a means by which alphaviruses may evade innate immune detection and activation prior to viral gene expression. Altogether, these data identify novel capsid protein–protein interactions, establish the capsid–IRAK1 interaction as a common alphavirus host–pathogen interface, and delineate the molecular consequences of the capsid–IRAK1 interaction on IRAK1-dependent signaling.

## 1. Introduction

Alphaviruses are positive-sense RNA viruses which are primarily spread via vector-competent mosquito species [1]. Collectively, and often on a seasonal basis, the members of genus *Alphavirus* are responsible for local, regional, and global outbreaks of clinically severe illness [2,3,4,5,6,7,8,9,10,11,12,13,14]. Alphaviruses may be broadly classified via their predominant symptomology as either arthritogenic or encephalitic. Arthritogenic alphaviruses, such as Sindbis virus (SINV; the prototypic alphavirus), Ross River virus (RRV), Semliki Forest virus (SFV), and Chikungunya virus (CHIKV), exhibit low mortality despite often causing febrile illness with debilitating multifocal arthritis [15,16,17,18,19,20,21,22,23]. In some instances, the multifocal arthritis may persist for several months to years past the resolution of the primary infection [15,16,17,24,25,26]. In contrast to arthritogenic alphaviruses, encephalitic alphaviruses can exhibit significant mortality and life-altering neurological sequelae, primarily in young children and adolescents [27,28,29,30]. Despite the clear impact of alphaviruses on global human health and quality of life in developing and developed communities alike, there are no clinically proven antiviral therapeutics, or safe and effective vaccines to mitigate the public health threat posed by alphaviruses. 

An infectious alphavirus particle is relatively simple in design. Measuring approximately 70 nm in diameter, an alphavirus particle features an RNA cargo surrounded by two concentric icosahedral protein layers divided by a host-derived lipid envelope [31,32]. The viral glycoproteins E1 and E2 (and in some instances E3) are ordered in an icosahedral array projecting from the external surface of the viral envelope [31,32]. Several copies of the viral 6K and TF proteins, precisely how many is unknown at this point, are associated with the viral envelope [33,34]. The *C*-terminal endodomain of the E2 protein interacts with the viral capsid (CP) protein, which also forms an icosahedral structure which is symmetrically aligned with the viral glycoprotein spikes. The CP protein is the sole viral protein component of the nucleocapsid core and is the most abundant viral protein in the mature viral particle [1]. The alphaviral entry pathway initiates with the interaction of the host receptor with the viral E2 glycoprotein, resulting in the endocytosis of the viral particle, and culminates with the delivery of the nucleocapsid core to the host cytoplasm [1]. The nucleocapsid core then rapidly disassembles, releasing the CP protein from the viral genomic RNA, the latter of which interacts with host factors to engage the translational machinery to initiate the synthesis of the viral replicase complex. 

While still becoming better understood at the molecular level, the fate of genomic RNA is straightforward. In contrast, despite being the predominant viral component released to the host cytoplasm, the role of the viral CP protein after entry is less understood. Prior work by the Sokoloski lab identified a series of non-assembly CP–RNA (naCP–RNA) interactions which functioned during the early stages of viral infection [35]. The disruption of the naCP–RNA interactions negatively impacted viral particle infectivity, which correlated with decreased viral RNA stability in cellular models of infection [35]. Collectively, these data led us to hypothesize that the alphaviral CP proteins which are delivered as part of the nucleocapsid core may function to influence the host cell environment after disassembly. We further postulated that the molecular activities of the CP protein are dependent on the formation of host–pathogen protein–protein interactions which impart new functionality to the CP protein complex, or disrupt the activities of the normal cellular protein complexes. 

The above overarching hypotheses, and the absence of a comprehensive analysis of the alphavirus CP protein–protein interaction data in the knowledgebase, led us to examine the protein–protein interactions of the SINV CP protein using an innovative approach. Here we detail the use of an adapted BioID approach to identify the putative host–pathogen interactions of the SINV CP protein [36,37]. This discovery approach led to the identification and validation of a novel alphaviral host–pathogen interaction—the interaction of the alphaviral CP protein with the host IRAK1 protein. The host IRAK1 protein is a critical component of the TLR and IL-1R signal transduction pathways, and thus the CP–IRAK1 interaction may negatively impact the detection and response to TLR and IL1R ligand binding [38,39]. Using a robust series of state-of-the-art model systems, we assessed the impact of the CP–IRAK1 on IRAK1-dependent signaling and found that that the alphaviral CP protein was capable of significantly inhibiting IRAK1-dependent TLR signaling. Importantly, the SINV CP proteins delivered from viral particles during viral entry were sufficient to mask TLR agonist detection, regardless of viral particle infectivity. Taken together, the data presented in this study significantly contribute to the field by i) using an unbiased approach to identify putative CP–protein interactions, and ii) delineating a novel mechanism by which the host innate immune response is evaded during the earliest stages of alphaviral infection prior to viral gene expression. 

## 2. Materials and Methods

### 2.1. Tissue Culture Cells

HEK293 (ATCC CRL-1573) and BHK-21 (ATCC CCL-10) cells were cultured in Minimal Essential Media (MEM; Cellgro Mediatech, Inc, Manassas, VA USA), supplemented with 10% Fetal Bovine Serum (FBS; Corning, Corning, NY USA), 1× Penicillin/Steptomycin (Pen/Strep; Corning, Corning, NY USA), 1× Non-Essential Amino Acids (NEAA; Corning, Corning, NY USA), and l-glutamine (Corning, Corning, NY USA). HEK293-derived reporter cells, namely HEK-Blue hTLR3, HEK-Blue hTLR4, and HEK-Blue hTLR7 (Invivogen, San Diego, CA USA), were cultured in Dulbecco’s Modified Eagle Medium (DMEM; Corning, Corning, NY USA) supplemented with 4.5 g/L glucose, 10% FBS, 1× Pen/Strep, and 1× Normocin (Invivogen, San Diego, CA USA). To maintain genetic homogeneity, the HEK-Blue tissue culture cells were maintained at low passage number and supplemented with the appropriate selection antibiotics on alternating passages to maintain genomic integrity (as indicated by Invivogen’s instructions per each cell line). All cell lines were cultured in humidified tissue culture incubators at 37 °C in the presence of 5.0% CO_2_.

### 2.2. Plasmids

The vertebrate expression plasmids for the BioID2 screen were independently constructed, but based off those previously utilized by Kim et al., 2016 [36]. Specifically, the pBioID2-Only, and pSINVCP-BioID2 plasmids were generated via the Gibson Assembly of DNA fragments encoding a cMyc-tagged BioID2 biotin ligase and the CP protein from SINV (strain AR86) into the pCDNA3.1/Zeo(+) expression vector. To enhance the stability of the SINV CP-BioID2 fusion protein, the protease activity of the alphaviral CP proteins was eliminated by the mutation of an essential active residue required for protease activity [40].

The vertebrate expression plasmids utilized in the Nanoluc BiMolecular Complementation studies described here were independently constructed, but based on those previously identified by Mo et al., 2017 [41]. Briefly, the Nanoluc protein was subdivided into two complementing fragments followed by a poly-glycine linker. N67, which consisted of the *N*-terminal 67 amino acids of the Nanoluc protein, and C67, which consists of the remaining amino acid residues, were subcloned via Gibson Assembly reactions into the vertebrate expression vector pCDNA3.1/Zeo(+). The resulting plasmids, pSplit.Nanoluc.N67 and pSplit.Nanoluc.C67, were then used in further Gibson Assembly reactions to create the plasmids used in this study. Briefly, these included pSplit.Nanoluc.N67.huIRAK1, which included the full-length human IRAK1 ORF, and the pSplit.Nanoluc.C67.SINV CP; RRV CP; EEE CP; VEE CP; CHIKV CP; and YFV CP plasmids which contained the full-length ORFs of the respective alphaviral or flaviviral capsid proteins. As above, to ensure the stability of the Nanoluc fragment fusion proteins, the protease activity of the alphaviral CP proteins was eliminated by the mutation of an essential enzymatically active residue required for protease activity [40]. Control plasmids including the BioID2 ORF in lieu of either the IRAK1 or the CP proteins were generated as non-specific controls. 

To express the native SINV CP protein in a context outside of SINV infection, a vertebrate expression plasmid encoding the wild-type SINV CP protein and a mCherry reporter was generated via Gibson Assembly into the pCDNA3.1/Zeo(+) vector. Specifically, the native ORFs of the SINV CP protein and E3 protein were fused to an mCherry ORF fragment to generate pEXPR.SINVCP.mCherry, which upon transfection into a cell will direct the synthesis of a CP-E3-mCherry polyprotein which is processed into CP and E3-mCherry via the native protease activity of the SINV CP protein. 

All DNA fragments for the generation of the clones above were synthesized by Genscript (GenScript USA Inc., Piscataway, NJ, USA) and assembled using the Gibson Assembly mastermix available from Synthetic Genomics, Inc. (Codex DNA Inc., San Diego, CA, USA) according to the manufacturer’s instructions. All plasmids were verified by sequencing prior to their use in these studies. Specific plasmid information, including details regarding the restriction enzymes used for their construction; antibiotic resistance markers and bacterial growth conditions; and complete plasmid sequences, are available upon direct request. 

All plasmids were cultured overnight in *E. coli* DH5α (or comparable) bacteria under antibiotic selection and purified via miniprep or midiprep purification kits (Omega Bio-Tek Inc., Norcross, GA, USA). The purified plasmid DNA was phenol chloroform extracted twice to remove any trace endotoxin or bacterial proteins from the plasmid preparations.

### 2.3. Generation and Preparation of SINV

This study utilized p389_P726G_, a Toto1101-derived SINV strain which encodes an EGFP reporter protein fused to the nsP3 gene [42], and a point mutation in the nsP2 protein which abrogates the inhibition of cellular transcription (P726G) [43]. The corresponding infectious clone of this SINV construct was generated via site-directed mutagenesis of the p389 infectious clone. This particular strain was chosen as it enables the rapid visual confirmation of viral infection and allows for continued cellular transcription during infections of highly permissive tissue culture cells. Infectious viral stocks were generated via the electroporation of in vitro transcribed RNA into BHK-21 cells, as previously described [44]. Briefly, ~3 × 10^6^ BHK-21 cells were electroporated with 10 µg of in vitro transcribed RNA using a single pulse at 1.5 kV, 25 mA, and 200 Ω. After the total infection of the monolayer (as determined by EGFP signal), the tissue culture supernatants were harvested and titered to determine the number of EGFP positive focus-forming units per ml using standard plaque assays. 

For the studies utilizing non-infectious SINV particles, the aforementioned SINV reporter mutant virus was inactivated via UV irradiation [45,46]. Briefly, 1 mL of virus stock was aliquoted into one well of a 24-well plate, on ice, and irradiated by exposure to 260 nm UV light in a Stratalinker for 5 min. The virus was promptly used, and any remaining inoculum was discarded. The verification of UV inactivation was accomplished via the visualization of no EGFP signal in inoculated BHK-21 cell monolayers after 24 h of infection.

### 2.4. TLR Agonists and Other Receptor Ligands

All agonists and recombinant protein ligands were diluted in pyrogen-, endotoxin-, and nuclease-free phosphate-buffered saline, or distilled water, as indicated below. The reconstituted agonists/ligands were aliquoted into single-use tubes and stored at −80 °C until use. The HEK293 TLR3 cells used in this study were stimulated with high-molecular-weight poly(I:C) (Invivogen, San Diego, CA, USA) diluted in 1× PBS. Prior to use, the poly(I:C) was heated to 75 °C and allowed to slow cool to room temperature to anneal the poly(I) and poly© RNA strands into dsRNA. The HEK293 TLR4 cells were stimulated with Kdo2-lipid A (Avanti Polar lipids) diluted in sterile nuclease free distilled water. Prior to use, the Kdo2-lipid A was sonicated to ensure a homogenous solution prior to aliquoting and storage. The HEK293 TLR7 cells were stimulated with CL307 (Invivogen, San Diego, CA, USA) diluted in sterile nuclease free distilled water. All of the HEK293 cells utilized in this study expressed native levels of TNFR receptors and were naturally responsive to stimulation with recombinant hTNFα (R&D Systems Inc., Minneapolis, MN, USA).

### 2.5. The Identification of SINV CP Protein–Protein Interactions

To identify the protein–protein interactions of the SINV CP protein, we utilized a modified method derived from the previously reported BioID screens [36,37,47,48]. Per purification, approximately 2 × 10^6^ HEK293 cells were cultured as 80% confluent monolayers under normal conditions prior to transfection with either pBioID2-Only, or pSINVCP-BioID2. Four hours after transfection, the tissue culture medium was replaced with fresh whole growth medium supplemented with 1 µM biotin. After a 24 h labeling incubation period, the tissue culture cells were washed with 1xPBS, and whole-cell lysates were generated via the addition of Lysis Buffer (50 mM Tris, pH 7.6; 500 mM NaCl; 0.4% Sodium Dodecyl Sulfate (SDS); 1 mM DiThioThreitol (DTT); and 2.0% Triton X-100). The whole-cell lysates were vortexed and frozen to ensure complete lysis, and the lysates were stored at −80 °C until ready for further use. 

To verify that the BioID2 biotin ligase was working during our discovery approach, and to confirm that the biotin labeling was specific, we assessed the whole-cell lysates using SDS-PAGE and Western Blotting techniques. Briefly, equal amounts of whole-cell lysates were resolved via 8% SDS-PAGE and blotted to PVDF membranes. The blotted proteins were then probed for protein biotinylation using streptavidin-HRP, or with anti-cMyc monoclonal antibodies to detect the individual expressed BioID2 fusion proteins.

To purify the biotinylated host proteins, the whole-cell lysates were thawed on ice prior to being vigorously vortexed and clarified via centrifugation at 16,000× *g* for 5 min. The clarified whole-cell lysates were transferred to a fresh microfuge tube and incubated with magnetic streptavidin beads for one hour at room temperature on a rotisserie mixer. After binding, the supernatant was removed and discarded, and the magnetic beads were washed 5 times to remove unbound proteins and non-specific contaminants. The bound proteins were then released from the streptavidin resin via resuspension in 6× Laemmli buffer and a 15 min incubation at 95 °C. 

The eluted proteins were transferred to a fresh microfuge tube and precipitated with 100% (*w*/*v*) TriChloroactic Acid (TCA) at a ratio of 1:4 (TCA:Sample). After vortexing, the samples were incubated on ice for 10 min to allow complete precipitation of the macromolecules in the solution. The precipitated proteins were pelleted via 5 min of centrifugation at 14,000× *g*, and the supernatant was decanted into an appropriate waste container. The protein pellet was washed three times with ice cold acetone, and the pellet was dried by incubating the microfuge tube at 95 °C for 5 min to drive off excess acetone.

The dried samples were resuspended in a minimal volume of 200 mM triethylammonium bicarbonate solution (pH 8.5) and reduced via the addition of 25 mM DTT stock solution to a final concentration of 5 mM. The samples were incubated at 65 °C for 30 min, prior to the addition of Iodoacetamide to a final concentration of 10 mM and further incubation at room temperature in the dark for a period of 30 min. The alkylated samples were digested via the addition of 0.1 µg of mass spec grade trypsin and incubation at 37 °C for 30 min. After the primary digestion period, a second bolus of 0.1 µg of trypsin was added, and the samples were further digested overnight at 37 °C. After digestion, the samples were dried in a SpeedVac and stored at −80 °C.

### 2.6. The Identification of Putative SINV Capsid Interactions by Mass Spectrometry

Prior to liquid chromatography and mass spectrometry, the dried samples were dissolved in 20 μL of 2% (*v*/*v*) acetonitrile and 0.1% (*v*/*v*) formic acid, and 2 μL of each sample was analyzed further. The columns used for liquid chromatography separation were an Acclaim PepMap 100 75 µm × 2 cm, nanoViper (C18, 3 µm, 100 Å) trap, and an Acclaim PepMap RSLC 75µm × 50 cm, nanoViper (C18, 2 µm, 100 Å) separating column heated at 50 °C. An EASY-nLC 1000 UHPLC system was used with solvents A = 2% (*v*/*v*) acetonitrile/0.1% (*v*/*v*) formic acid, and B = 80% (*v*/*v*) acetonitrile/0.1% (*v*/*v*) formic acid. Following injection onto the trap, the sample was separated with a 165 min linear gradient from 0% to 55% B at 250 nL/min, followed by a 5 min linear gradient from 55% to 95% B with a flow ramp from 250 to 300 nL/min, and lastly a 10 min wash with 95% B at 300 nL/min. A 40 mm stainless steel emitter was coupled to the outlet of the separating column. A Nanospray Flex source was used to position the end of the emitter near the ion transfer capillary of the mass spectrometer. The ion transfer capillary temperature was set at 225 °C, and the spray voltage at 1.75 kV.

An Orbitrap Elite ETD mass spectrometer was used to collect data from the LC eluate. An Nth-Order Double Play was created in Xcalibur v2.2. Scan event one obtained an FTMS MS1 scan (normal mass range, 240,000 resolution, full scan type, positive polarity, profile data type) for the range 300–2000 m/z. Scan event two obtained ITMS MS2 scans (normal mass range, rapid scan rate, centroid data type) on up to twenty peaks that had a minimum signal threshold of 5000 counts from scan event one. Either the lock mass option was enabled (0% lock mass abundance) or RAW files were recalibrated offline in Xcalibur v2.2 using the 371.101236 m/z polysiloxane peak as an internal calibrant.

Proteome Discoverer v1.4.1.14 was used to analyze the data. The 9/27/2018 version of the UniprotKB reviewed reference proteome canonical and isoform Homo sapiens sequences (Proteome ID UP000005640) concatenated with BioID2 and SINV capsid BioID2 sequences was used in the Mascot v2.5.1 and SequestHT searches. The enzyme specified was trypsin (maximum two missed cleavages with inhibition by P) with Carbamidomethyl(C) as a static modification and Oxidation(M), Biotin(K) as dynamic. Fragment tolerance was 1.0 Da (monoisotopic) and parent tolerance was 50 ppm (monoisotopic). A Target Decoy PSM Validator node was included in the Proteome Discoverer workflow.

The result files from Proteome Discoverer were loaded into Scaffold Q+S v4.4.5. Scaffold was used to calculate the false discovery rate using the Scaffold Local FDR and Protein Prophet algorithms. Peptides were accepted if the identification had a probability greater than 99.9% and a parent mass error within 10 ppm. Proteins were accepted if they had a probability greater than 99.9% and at least two peptides. Proteins were grouped into clusters to satisfy the parsimony principle.

The host proteins identified by the BioID2 approach were assigned as specific or non-specific on the basis of their relative detection in the BioD2-CP or BioID2 Control mass spectrometry data sets. To reduce the introduction of bias in the data sets, any relative peptide quantification data were disregarded, and proteins were considered duly identified if uniquely assignable peptides were detected.

### 2.7. Bioinformatic/Ontological Analyses of Putative SINV CP Protein–Protein Interactions

To identify whether or not the host proteins identified by the BioID2 discovery approach were subject to unintentional bias on the basis of their relative protein abundance in the host proteome, we compared the relative protein abundances of the non-specific and SINV CP-specific data sets to the HEK293 proteome [49]. 

The 19 host factors identified by the SINV capsid BioID2 discovery approach as specific to the SINV CP protein were examined using the STRING analysis (version 11.0) algorithm to detect the presence of protein–protein interaction networks [50]. The parameters used to define the presence or absence of interaction networking included gene fusion, co-occurrence, experiments, databases, and text mining, and the confidence level was set to medium. The confidence/strength of interactions between individual host factors were scaled (arbitrarily by STRINGS version 11.0) and indicated via line weight between interconnected nodes, with higher weight indicating greater confidence. 

In addition to the identification/visualization of interaction networks, the 19 putatively identified interactants were examined ontologically using DAVID to identify enriched cellular component, molecular function, and biological process ontological groups [51,52]. Due to the relatively small number of host proteins in the specific group, the fold enrichment, and relative statistical significances of any identified ontological groups exhibited considerable range (as noted in the text).

The entire BioID2 data sets and ontological analyses are available as supplemental data files (see Supplemental File S1 in the accompanying supplemental materials).

### 2.8. Nanoluciferase-Based BiMolecular Complementation Analysis (Nanoluc BiMC)

To validate the interaction between IRAK1 and SINV CP, we utilized an innovative BiMC approach to overcome the non-specific interaction of the CP proteins with purification resins [41]. In these experiments, HEK293 cells were seeded into flat white-bottommed 96-well plates at a density of 1.25 × 10^4^ cells per well. After an overnight incubation period, the cells were co-transfected with pSplit.Nanoluc.C67 plasmids encoding either an alphaviral CP protein or the BioID2 protein as a control, and the corresponding pSplit.Nanoluc.N67 plasmid encoding the human IRAK1 protein using Lipofectamine 3000 (Invitrogen, Thermo Fisher Scientific, Waltham, MA, USA). Specific transfection conditions for the Nanoluc BiMC assay consisted of 50 ng of each expression plasmid to achieve a total of 0.1 µg of DNA. The cells were transfected in whole growth media and incubated for a period of 48 h under normal conditions prior to the assessment of Nanoluc complementation via the quantitative detection of Nanoluc activity via live-cell NanoGlo reagents.

Briefly, to measure the levels of Nanoluc activity the growth medium was gently removed and replaced with 100 μL of Optimem media (Thermo Fisher Scientific, Waltham, MA, USA). Immediately after the addition of the Optimem media, NanoGlo live-cell assay (Promega, Madison, WI, USA) reagents were prepared fresh as according to the manufacturer’s instructions, and rapidly added to each well. The plate was briefly rocked by hand to ensure the Nanoglo reagent and cell culture media were well mixed prior to the detection of luminescence in a Synergy H1 microplate reader.

### 2.9. Quantitative Analysis of TLR, IL1R, and TNFR Signaling

Aside from the obvious differences in regards to the agonists/ligands being utilized, the overall experimental approaches used to determine the impact of the CP–IRAK1 interaction were identical. For all assays, the HEK293-derived reporter cell lines were cultured to ~75% confluence in a 96-well format in whole media lacking antibiotic selection prior to being experimentally treated and assessed as follows.

To determine the impact of the SINV CP protein on IRAK1-dependent signaling, the HEK293-derived reporter cell monolayers were transfected with expression plasmids encoding either the BioID2 control plasmid or a SINV CP-E3-mCherry fusion protein capable of producing the native full-length SINV CP protein after cleavage from the C-terminal E3-mCherry fusion protein. At 24 h post transfection, the supernatant was removed and replaced with whole growth medium supplemented with the indicated receptor agonists/ligands, and the cells were returned to the incubator for a period of 16 h. After the agonist/ligand activation period, the tissue culture supernatants were harvested and assayed as described below.

To determine the impact of SINV infection on IRAK1-dependent signaling, the HEK293-derived reporter cell monolayers were either mock infected or infected with SINV_P726G_ at an MOI of 10 PFU/cell. Twelve hours post infection, the tissue culture media was removed and replaced with fresh pre-warmed whole growth medium supplemented with the indicated receptor agonists/ligands, and the tissue culture cells were returned to the incubator and incubated under normal conditions for a period of 16 h. After the agonist/ligand activation period, the tissue culture supernatants were harvested and assayed as below.

To determine the impact of SINV co-exposure on IRAK1-dependent signaling, we modified the above approach. Specifically, the HEK293-derived reporter cell monolayers were either mock infected or infected with SINV_P726G_ at an MOI of 10 PFU/cell in media supplemented with the aforementioned receptor agonists/ligands for a period of 1 h at 37 °C in a 5.0% CO2 tissue culture incubator. After the co-exposure period, the treatment media was removed and the monolayers gently washed with pre-warmed whole growth medium to remove residual virus/ligand. A minimal volume of whole growth medium was added to the cell monolayers, and the cells were incubated for 16 h prior to harvesting the tissue culture supernatants for assaying. 

To define whether or not SINV replication/gene expression was required for the inhibition of IRAK1-dependent signaling, the co-exposure experiment described above was performed identically with the exception that UV inactivated SINV particles were utilized. Similarly, to determine whether delivery of the nucleocapsid core to the host cytoplasm was required for the disruption of IRAK1-dependent signaling, the aforedescribed co-exposure experiments utilizing infectious SINV were performed in the presence of whole growth medium supplemented with 40 µM ammonium chloride to block the final steps of the viral entry pathway by preventing acidification of the endosome. 

For all of the experimental approaches described above, the harvested tissue culture supernatants were immediately quantitatively assayed for the presence of Secreted Embryonic Alkaline Phosphatase via the use of QuantiBlue detection medium (Invivogen, San Diego, CA, USA). Briefly, in a sterile clear-bottomed 96-well plate, 20 μL of cell-free tissue culture supernatant was added to 180 μL of QuantiBlue detection reagent and the solutions were mixed by gentle pipetting. Afterwards, the 96-well plate was incubated at 37 °C in a plate reader, and absorbance readings at 620 nm were taken regularly for a period of three hours, or until the A620 nm curves of the highest agonist concentrations indicated saturation of the limit of detection. The A620 nm readings from pre-saturation time points were comparatively assessed to determine agonist/ligand detection via the level of NFĸB activation as determined by the SEAP assay colorimetric readout. 

The quantitative analysis of signal transduction, as per NFĸB activation, was determined by comparing the SEAP activity of the control and experimental conditions over the agonist dose range after the subtraction of non-agonist-treated wells [53,54]. Specifically, the control agonist treatment with the highest level of relative SEAP activity within the given dose range was standardized to 100%, and all other wells were normalized accordingly to determine their relative SEAP activity to the identified maximum observed value. The quantitative data obtained from multiple biological replicates for a given dose concentration were averaged and plotted with respect to agonist concentration. Non-linear regression analysis of the data, via GraphPad Prism 7.0.2 using the log(agonist) vs. response variable slope (four parameters) non-linear curve fit function, was used to determine the activation profiles in response to agonist treatment, and the 95% confidence intervals of the data. In addition, the agonist concentrations at which the control and experimental treatments reached 50% maximal activity (EC50_MAX_) were determined using these non-linear regression calculations.

### 2.10. Statistical Analyses

The quantitative data reported in this study represent the means of at least 5 biological replicates from at least two independent viral stocks, or DNA plasmid preparations, as specifically indicated in the figure legends. The error bars for any given quantitative value represent the standard deviations of the means. The statistical analysis of comparative samples was accomplished using variable bootstrapping, as previously described [44]. Any *p*-values for a given data set were determined via one-way ANOVA analyses and reconfirmed using Student’s *t*-test as a post hoc analysis. Bioinformatics analyses were completed using the standard analyses of the STRING analysis (version 11.0) and DAVID gene ontology informatics suites, as described in the text.

## 3. Results

### 3.1. The Discovery of Novel Sindbis Virus Capsid Protein–Protein Interactions

Previous work from our lab demonstrated that the SINV CP protein binds to the SINV viral genomic RNA at discrete interaction sites to accomplish non-assembly associated roles during infection. Further characterizations indicated that when the non-assembly SINV CP–RNA interactions were disrupted the incoming genomic vRNAs had significantly decreased half-lives relative to wild-type SINV RNAs [35]. This led to the speculation that the non-assembly CP–RNA interactions were involved in the regulation of viral genomic RNA stability early during infection following the disassembly of the nucleocapsid core. Nonetheless, we postulated that the SINV CP protein was unlikely capable of directly mediating RNA stability by itself; and thus, we set out to define the extent to which the SINV CP protein engaged with host factors via protein–protein interactions. 

**Figure 1:**
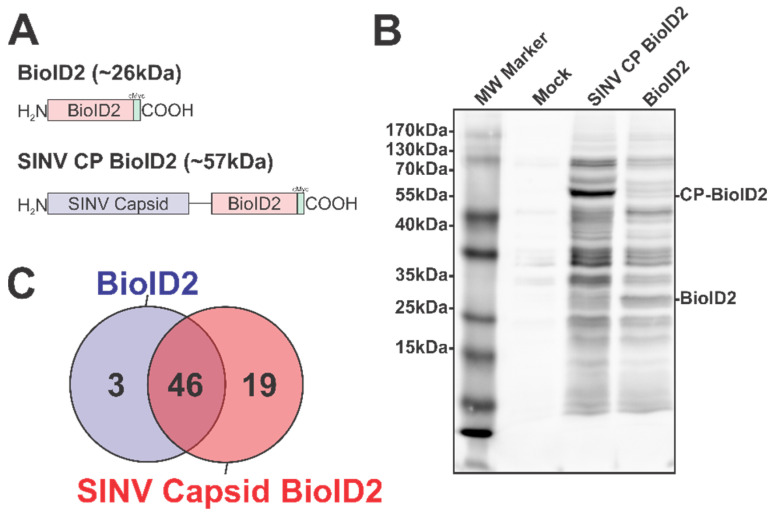
The Identification of the Host–Pathogen Interactions of the SINV Capsid Protein. (**A**) A diagram of the BioID2 fusion proteins expressed in 293HEK cells via plasmid transfection. Individual domains are labeled above. The line in the SINV CP BioID2 construct represents a poly-glycine linker, and the green box represents a cMyc tag. (**B**) A representative blot of 293HEK cell lysates after the BioID2 approach. Briefly, transfected or control transfected cells were cultured in the presence of excess biotin prior to the generation of whole-cell lysates. Equal protein amounts were resolved using SDS-PAGE, and subsequently probed for protein biotinylation using streptavidin-HRP. (**C**) A Venn diagram of the host proteins identified by mass spectrometry after the BioID2 approach designated the host factors as either non-specific or specific to either BioID2 transfection/purification.

To overcome the challenges associated with working with the alphaviral CP proteins, we adapted the BioID2 discovery approach to identify SINV CP host interactions in an unbiased manner [36,37,47]. In this approach, the expression of BioID2 fusion proteins in the presence of excess biotin results in the labeling of protein interactants, allowing for subsequent affinity purification and identification via mass spectrometry. As depicted in Figure 1A, we fused the coding region of the promiscuous BioID2 biotin ligase to the C-terminus of the SINV CP protein in a mammalian expression plasmid, thereby enabling the ectopic expression of a BioID2-CP fusion protein after the transfection of the BioID2-SINV CP expression plasmid in to HEK293 cells. 

To test the functionality of the BioID2 biotin ligase after fusion to the SINV CP protein, whole-cell lysates were generated from HEK293 cells transfected with either the BioID2-CP or BioID2-Control expression plasmids, or mock transfected, following incubation in the presence of excess biotin. Equal amounts of whole-cell lysate were resolved via SDS-PAGE and transferred to PVDF prior to being probed for protein biotinylation using HRP-conjugated streptavidin (Figure 1B). As shown by the presence of readily detectable protein species in the BioID2-CP and the BioID2-Control lanes, and the relative absence of signal in the mock-treated lane, the BioID2 biotin ligase was functional when fused to the SINV CP protein. Importantly, the overall labeling patterns of the BioID2-CP and BioID2-Control lanes exhibited unique profiles relative to one another, suggesting that the fusion of the CP protein to the BioID2 biotin ligase resulted in the specific labeling of putative CP interactants. Subsequent Western blotting with anti-Myc tag monoclonal antibodies revealed that the major protein species in either BioID2-containing transfection condition were the ectopically expressed BioID2 fusion proteins themselves and confirmed that none of the other high-molecular-weight species were BioID2-CP truncation products. 

As the functionality of the BioID2-CP fusion approach had been confirmed, we next wanted to identify the host factors which engaged with the SINV CP protein during BioID2-CP expression. To this end, we transfected the aforementioned BioID2 expression plasmids into HEK293 cells and generated whole-cell lysates on a preparative scale for identification of putative interactants by mass spectrometry. As briefly described above, the biotinylated protein species from BioID2-CP and BioID2-Control whole-cell extracts were purified using streptavidin resin prior to the development of trypsin digested peptide libraries for high sensitivity mass spectrometry. 

In total, the two independent BioID2-CP data sets had a total of 85 and 90 unique proteins identified; whereas the BioID2-Only control had 59 and 79 unique proteins identified. Comparative analysis of the mass spectrometric data arising from two independent BioID2-CP and BioID2-Control purifications was used to identify and assign interaction specificity to putative interactants. To ensure a high degree of rigor during the discovery approach in order to be assigned as a SINV CP protein interactant a given host protein had to be detected in both of the SINV CP data sets, and absent in either of the BioID2-Control data sets. Similarly, in order to be considered a “genuine” non-specific BioID2 interactant, a given host protein had to be reproducibly detected in both BioID2-Control data sets. As shown in Figure 1C, these comparative analyses revealed that a total of 68 proteins were assignable as identified interactants. Of these, 46 were identified as common between the BioID2-CP and BioID2-control, and 3 were present solely in the BioID2-Control samples, leaving 19 proteins unique to BioID2-CP (Figure 1C). 

Altogether, these data confirm that the BioID2 approach represents a means by which the host–pathogen interactions of the alphaviral CP proteins may be elucidated in a manner unrestricted by cross-linking or co-translational labeling kinetics. These efforts have led to the identification of 19 putative CP–protein interactions.

### 3.2. Ontological Analyses Reveal Novel Host–Pathogen Interactions

**Figure 2:**
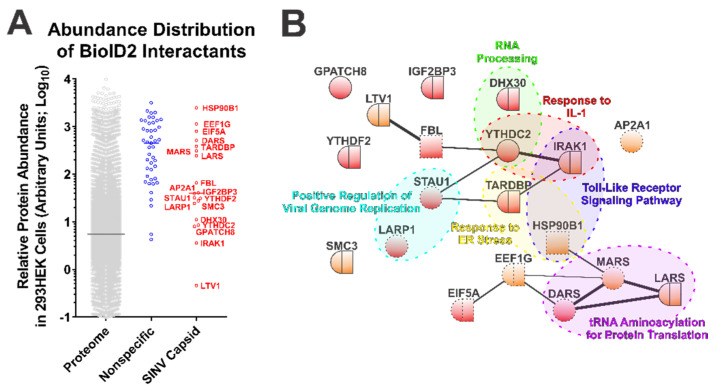
Ontological Analysis of the SINV CP–Protein Interactants Reveals Novel Host–Pathogen Interfaces. (**A**) Comparative analysis of arbitrary protein abundance of the host proteome (293HEK), and the non-specific and CP-specific interactants identified via the BioID2 approach. The lines on the graph represent the median abundance within the given data set, and the CP-specific interactants are indicated next to their corresponding data point. (**B**) A STRINGs interaction network map of the CP-specific interactants. The color and styling of the individual nodes indicates the properties of the corresponding protein as determined by ontological categorization: round = cytoplasmic localization; square = nuclear localization; round/square = shuttling protein, or found in both compartments; red = RNA-associated protein; dashed outline = membrane associated. The weight of the linear connections between the individual nodes is indicative of the relative strength/confidence of the interaction. Molecular function ontological groups, as described in depth in the text, are highlighted in a color-coded manner.

While the BioID2-CP screen led to the identification of novel SINV CP protein–protein interactions, interaction discovery screens are often subject to type-I errors. To determine the likelihood of a putative interactant being from a genuine CP–protein interaction and not the simple function of protein abundance, we compared the data obtained from the control and SINV CP BioID2 purifications with the relative protein abundances of the HEK293 proteome [49]. This analysis, while not directly evidentiary, enables a qualitative assessment of purity by identifying whether or not a set of interactants (or an individual interactant) may be over-represented on the simple basis of protein abundance. As presented in Figure 2A, the host factors detected and assigned as specific to the SINV CP conditions generally were of lower relative protein abundance than those identified and assigned as non-specific interactants. Nonetheless, several of the SINV CP-specific host proteins were comparable to the non-specific interactants in regards to their arbitrary abundances in the proteome. 

The 19 host factors detected during the above SINV CP BioID2 discovery approach were examined via the STRING protein–protein interaction network and functional enrichment analysis tool to identify common interaction networks and molecular/biological function ontologies [50,55]. As shown in Figure 2B, STRING analysis (version 11.0) revealed that several of the CP protein interactants exhibited protein–protein interactions with each other independent of the CP protein, suggesting possible indirect labeling of protein complexes. Nevertheless, the group of CP interactants at large was overall devoid of extensive interrelatedness, providing an indication that the SINV CP protein interacts with host factors in a largely specific manner. Ontological analyses provided further insight into the biological functions of the SINV CP interactants. As depicted in Figure 2B, analysis of cellular component ontology revealed that the putative interactants were associated with the cytosol (GO:0005829), the cytoplasm (GO:0005737), membranes (GO:0016020), and the nucleus (GO:0005634) to statistically significant degrees (all with *p*-values < 0.05, and all surviving post hoc Bonferroni analyses). However, the fold enrichments were ranged modestly from 2 to 4-fold. Analysis of molecular function indicated enrichment of the poly(A) RNA binding (GO:0044822), protein binding (GO:0005515), RNA binding (GO:0003723), ATP binding (GO:0005524), mRNA 3′-UTR binding (GO:0003730), and nucleic acid binding (GO:0003676) ontological groups. As with the analysis of cellular component ontology, each of the aforementioned groups were statistically significant by Fisher’s exact test (*p*-values < 0.05) and survived Bonferroni post hoc analyses (with the exception of mRNA 3′-UTR binding and nucleic acid binding), and enrichment ranged from 1.8 to 10-fold amongst the FDR correction survivors. 

Additionally, as highlighted in Figure 2B, several functional clusters were identifiable amongst the putative interactants identified by the BioID2-CP screen. Notable clusters of biological function with significant enrichment ( >15-fold) include the Positive Regulation of Viral Genome Replication (GO:0045070), RNA Processing (GO:0006396), Response to ER Stress (GO:0034976), tRNA Aminoacylation for Protein Translation (GO:0006418), Response to IL-1 (GO:0070555), and Toll-Like Receptor Signaling Pathway (GO:0002224). While the biological process GO terms listed had considerable enrichment, and initial statistical significance by Fisher’s exact test (with the exception of Response to ER Stress, and RNA Processing where the *p*-values were greater than the statistical threshold of 0.05), all GO clusters succumbed to false discovery rate adjustments (likely due to the relatively few numbers of proteins in each group). 

The above data indicate that the SINV CP protein is associated with a number of cytosolic RNA- and protein-binding proteins; however, these data do not indicate a singular extensive/monolithic role for the SINV CP protein in any particular cellular process outside of infection. The association of the CP protein with host factors involved in the stability of cellular RNAs is consistent with the aforementioned non-assembly roles of the SINV CP protein during infection. 

The detection of the host IRAK1 protein as a putative CP–protein interaction significantly drew our attention due to the critical roles of the IRAK1 protein in TLR and IL1R signaling [38,39]. Previous studies have demonstrated that the host TLRs contribute to the control of alphaviral infection, as MyD88−/− mice exhibited enhanced viremia and viral dissemination relative to wild-type controls [56,57]. Similarly, TLR7−/− mice exhibit increased pathology and viral burdens during alphaviral infections [58]. As such, given the impact of the TLRs on alphaviral infection, we focused our efforts on evaluating the CP–IRAK1 interaction at a greater level of molecular and biological depth.

### 3.3. The SINV CP–IRAK1 Interaction Is Genuine, and the CP–IRAK1 Interaction Is Conserved across the Genus Alphavirus

**Figure 3:**
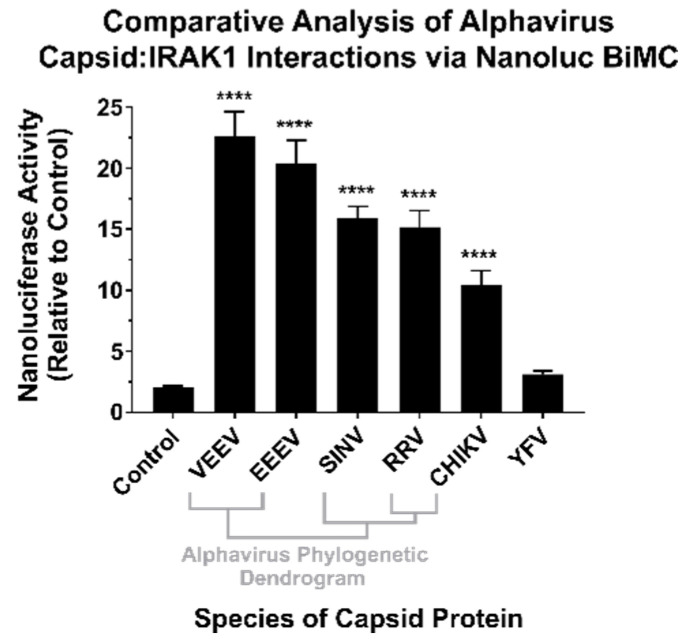
The CP–IRAK1 Interaction Is Genuine, and Widely Conserved across the Genus *Alphavirus*. The interaction of the alphaviral CP proteins with the host IRAK1 protein was assessed using Nanoluc-based BiMolecular Complementation (BiMC). Briefly, 293HEK cells were co-transfected with expression plasmids encoding the human IRAK1 protein and one of the indicated CP proteins or the BioID2 protein fused to complementary fragments of Nanoluc. Forty-eight hours post transfection the cells were assayed using the NanoGlo live-cell assay system, and the luminescence was detected using a plate reader. The luminescent intensity of the CP–IRAK1 BiMC conditions was compared relative to those of control reactions lacking an interacting pair of Nanoluc fragments. The quantitative data shown are the mean of at least five biological replicates, with the error bar representing the standard deviation of the means. Statistical significance relative to the control reactions, with a *p*-value of < 0.0001 = ****, was determined by one-way ANOVA analysis. Below the X axis is a phylogenetic dendrogram of alphavirus CP amino acid relatedness.

To confirm the results of the BioID experiments, we utilized a BiMolecular Complementation (BiMC) approach that utilized two fragments of the Nanoluc reporter [41]. Accordingly, the *N* terminal BiMC fragment of Nanoluc was fused to the human IRAK1 protein, and the complementary C-terminal fragment was fused to either the SINV, Chikungunya virus (CHIKV), Ross River virus (RRV), Venezuelan Equine Encephalitis virus (VEEV), Eastern Equine Encephalitis virus (EEEV), or Yellow Fever virus (YFV) capsid proteins, or the BioID2 protein as a control. To confirm and independently validate the observations of the BioID2 discovery approach, HEK293 cells were co-transfected with *N*-terminal Nanoluc IRAK1 plasmid and one of the above-mentioned complementary C-terminal expression plasmids. Forty-eight hours post transfection, the cells were treated with NanoGlo live-cell reagent and assayed for luminescence in a plate reader. As shown in Figure 3, co-expression of the IRAK1 and SINV capsid Nanoluc BiMC proteins resulted in significantly increased Nanoluc activity relative to the control reactions, with an approximately 12-fold difference between the two experimental conditions. Similarly, co-expression of the Nanoluc-IRAK1 protein with the other alphavirus CP proteins also significantly restored Nanoluc activity relative to the control. Specifically, the new world alphaviruses VEEV and EEEV demonstrated the highest BiMC activity with the human IRAK1 protein, exhibiting approximately 22-fold and 20-fold greater signal than control reactions, respectively. The CP proteins of the Old World alphaviruses RRV and CHIKV exhibited similar BiMC profiles to SINV, with greater than 10-fold Nanoluc activity relative to control reactions. 

Therefore, the CP–IRAK1 interaction is genuine and is conserved amongst multiple members of the genus *Alphavirus*. While the consequences of this interaction cannot be directly inferred from these data, we hypothesized a scenario in which the functionality of the IRAK1 protein was compromised by the CP–IRAK1 interaction. Given the importance of the IRAK1 protein to TLR and IL-1 signaling, the host’s capacity to respond to viral infection would be significantly perturbed if the CP–IRAK1 interaction suppressed the capacity of the IRAK1 protein to function.

### 3.4. The Sindbis Capsid Protein Is Sufficient to Inhibit IRAK1-Dependent Signaling

Following the validation of the SINV CP–IRAK1 interaction using BiMC, we hypothesized that the CP–IRAK1 interaction might be a means by which alphaviruses interfere with IRAK1-dependent signaling during infection to evade the induction of an antiviral innate immune response early during infection. To test this hypothesis, we utilized a series of robust/highly tractable tissue culture model systems which have been previously demonstrated to be effective in quantitatively assessing TLR activation [53,54,59,60,61,62]. Thus, we examined the impact of the SINV CP protein on IRAK1-dependent and independent signaling events using a series of 293HEK-based reporter cell lines which express Secreted Alkaline Phosphatase (SEAP) upon stimulation of TLRs 3, 4, and 7, or the TNFα receptor, via an NFκB/AP1-responsive promoter. 

**Figure 4:**
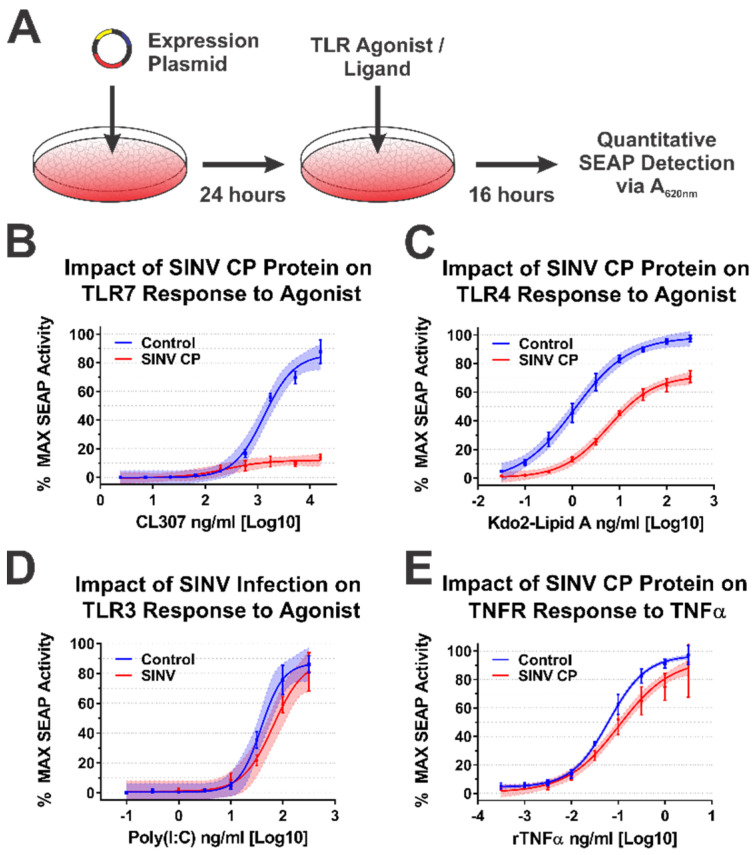
The SINV Capsid Protein Inhibits IRAK1-Dependent Signaling in a Specific Manner. (**A**) A diagram of the experimental approach used to test the capacity of the SINV CP protein to inhibit IRAK1-dependent signaling in a specific manner. Comparison of the curves in each panel reveals the impact of SINV CP protein expression on (**B**) TLR7 activation by CL307, (**C**) TLR4 activation by Kdo2-lipid A, (**D**) TLR3 activation by poly(I:C), and (**E**) TNFR activation by rTNFα. In all graphs, cells receiving control transfections prior to agonist treatment are represented by blue lines and data points, and those receiving the SINV CP protein expression plasmid are represented by red lines and data points. All quantitative data shown are the minimum of six independent biological replicates conducted over several days with at least two independent plasmid preparations. Quantitative data shown are the means of the biological replicates, and the error bars represent the standard deviation of the means. The connecting line represents a non-linear regression of the underlying data, and the shaded region indicates the 95% confidence interval of the non-linear regression. Thus, data points where the shaded regions do not intersect are statistically significant by at least a *p*-value of <0.05, as determined by ANOVA analysis.

Concisely, the aforementioned 293HEK reporter cell lines were transfected with mammalian expression vectors encoding either the SINV CP protein or the BioID2 protein. Twenty-four hours post transfection the culture medium was replaced, and the cells were treated with agonists appropriate for each target receptor over a broad dose range in half-log dilution steps. The agonist/ligand-treated cells were allowed to further incubate for 16 h post treatment (hpt) prior to the colorimetric assessment of SEAP activity in a plate reader (Figure 4A). As shown in Figure 4B, the ectopic expression of the SINV CP protein negatively impacted TLR7 signaling to a dramatic extent, as evidenced by a near complete loss of TLR activation over the CL307 agonist dose range examined relative to control treated cells. Similarly, albeit to a lesser extent, ectopic expression of the SINV CP protein negatively impacted TLR4 signaling, as evidenced by reduced maximal activation, and the amount of Kdo2-lipid A agonist required to reach an equivalent EC50_MAX_ response of the control cells was increased by >10-fold (Figure 4C).

To control for the possibility that the SINV CP protein was non-specifically interfering with cellular signaling and/or NFkB transcriptional activation, we examined the dose responsiveness of two IRAK1-independent signaling pathways after stimulation with their cognate receptor ligands during ectopic expression of the SINV CP protein. The TLR3 receptor is functionally unique amongst the TLRs in that it induces NFkB-mediated gene expression in an IRAK1-independent manner [63]. Hence, to determine whether the SINV CP protein non-specifically inhibited NFkB-mediated gene expression, we examined the dose responsiveness of TLR3 reporter cells to high-molecular-weight poly(I:C). Consistent with our hypothesis that the SINV CP protein interferes with TLR signaling in an IRAK1-dependent manner, the dose responsiveness of TLR3 was unaffected by the SINV CP protein (Figure 4D). Nonetheless, to further demonstrate that IRAK1-independent signaling and NFkB responsive transcription were not non-specifically perturbed in each of the cellular reporter systems utilized in this study, we examined the dose responsiveness of TNFα receptor (TNFR) to recombinant TNFα in each of the aforementioned cell lines during SINV infection. As depicted by the data in Figure 4E, the SINV CP protein did not pointedly interfere with TNRR signaling, as evidenced by similar EC50_MAX_ values despite statistically significant but quantitatively modest differences at the highest concentrations of agonist. However, these data may be driven by increased signal variation at the higher concentrations of rTNFα. Thus, the inhibitory effects observed for IRAK1-dependent signaling events are specific and not due to simple disruption of intracellular signaling or the inhibition of transcription/translation. 

**Figure 5:**
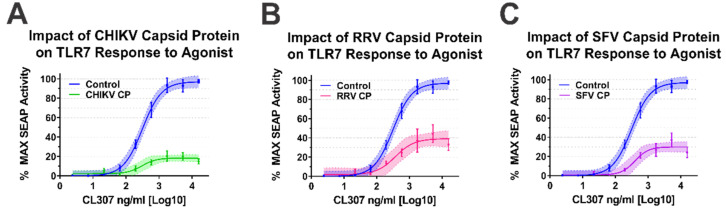
Old World Alphavirus Capsid Proteins Inhibit IRAK1-Dependent TLR7 Signaling. Comparison of the curves in each panel above reveals the impact of the Old World Alphaviral CP proteins expression on TLR7 activation by CL307. Specifically, the impact of ectopic expression of (**A**) CHIKV CP protein, (**B**) RRV CP protein, and (**C**) SFV CP proteins were assayed identically to that described for Figure 4. Quantitative data shown are the means of the biological replicates, and the error bars represent the standard deviation of the means. The connecting line represents a non-linear regression of the underlying data, and the shaded region indicates the 95% confidence interval of the non-linear regression. Thus, data points where the shaded regions do not intersect are statistically significant by at least a *p*-value of < 0.05, as determined by ANOVA analysis.

As our BiMC studies indicated that multiple alphaviral CP protein species interact with IRAK1, we sought to determine whether they impacted TLR7 activation. For simplicity, we focused our efforts on the Old World alphavirus species as their respective CP proteins are not known to negatively impact host transcription [64]. As shown in Figure 5, the ectopic expression of the CP proteins of CHIKV, RRV, and SFV all negatively impacted the capacity of the TLR7 receptor to respond to CL307 agonist treatment. 

Altogether, the data presented in Figure 4 and Figure 5 indicate that the alphaviral CP protein is sufficient and directly capable of inhibiting IRAK1-dependent signaling in a highly specific manner, strongly supporting our hypothesis that the CP–IRAK1 interaction represents a means by which the host innate immune response may be evaded during infection. The data presented in Figure 3 and Figure 5 are supportive of the conclusion that the CP–IRAK1 interaction is functionally conserved amongst several members of the genus. Nonetheless, the ectopic expression of the SINV CP protein likely does not directly mimic the levels of alphaviral CP protein generated during natural infections, or those delivered by the incoming viral particles. 

### 3.5. Sindbis Virus Infection Impairs IRAK1-Dependent Signaling in Tissue Culture Model Systems

While the data presented in Figure 4 and Figure 5 were highly supportive of our initial hypothesis that the SINV CP–IRAK1 interaction represents a means by which alphaviruses may evade the induction of the host innate immune response via the disruption of IRAK1-dependent signaling, the conditions assayed above do not mimic those of genuine viral infection. Indeed, the ectopic expression of the alphaviral CP proteins likely results in the overestimation of the impact of the interaction as the intracellular levels of the CP protein are likely to be in far excess of those observed during infection. To this end, we sought to define the impact of the SINV CP protein in a model system where the CP protein was derived from bona fide infectious events. 

Because a hallmark of alphaviral infection in highly permissive cells is the shutoff of host macromolecular synthesis, we employed a previously established approach which utilizes a SINV mutant that does not shut down host transcription, specifically the P726G point mutant of the SINV nsP2 protein, to enable SEAP activation in response to agonist treatment [43]. 

**Figure 6:**
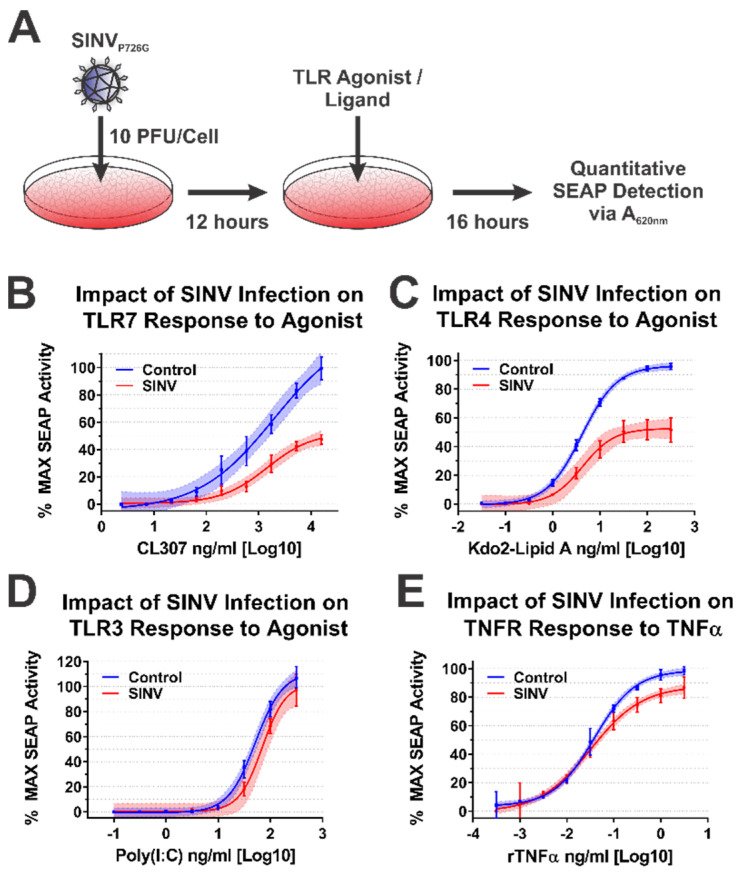
The SINV Infection Inhibits IRAK1-Dependent Signaling. (**A**) A diagram of the experimental approach used to test the capacity of SINV_P726G_ to inhibit IRAK1-dependent signaling in a specific manner during infection. Comparison of the curves in each panel reveals the impact of SINV infection on (**B**) TLR7 activation by CL307, (**C**) TLR4 activation by Kdo2-lipid A, (**D**) TLR3 activation by poly(I:C), and (**E**) TNFR activation by rTNFα. In all graphs, cells mock infected prior to agonist treatment are represented by blue lines and data points, and those receiving infectious SINV_P726G_ represented by red lines and data points. All quantitative data shown are the minimum of six independent biological replicates conducted over several days with at least two independent SINV preparations. Quantitative data shown are the means of the biological replicates, and the error bars represent the standard deviation of the means. The connecting line represents a non-linear regression of the underlying data, and the shaded region indicates the 95% confidence interval of the non-linear regression. Thus, data points where the shaded regions do not intersect are statistically significant by at least a *p*-value of < 0.05, as determined by ANOVA analysis.

To test the impact of the SINV CP protein on IRAK1-dependent signaling during SINV infection, we infected the battery of 293HEK-derived TLR reporter cells described above with a Toto1101-derived SINV GFP reporter strain that included the nsP2 P726G mutation at an MOI of 10 PFU/cell. The total infection of the cell monolayer was confirmed via GFP fluorescence, and 12 h post infection (hpi) the culture medium was replaced, and the cells were treated with agonists appropriate for each target receptor over as described above. The agonist/ligand-treated cells were further incubated for 16 h post treatment (hpt) prior to the colorimetric assessment of SEAP activity in a plate reader (Figure 6A). 

Consistent with the data presented above, SINV infection significantly impaired IRAK1-dependent signaling events, as demonstrated by decreased maximal activation and dose responsiveness to agonist treatment for SINV infected cells relative to mock infected controls. Specifically, as shown in Figure 6B, TLR7 maximal activation and dose responsiveness were reduced by 2-fold, and ~50-fold, respectively. TLR4 activation was similarly impacted, as TLR4 reporter cells infected with SINV exhibited a ~2-fold decrease in maximal activation relative to mock infected cells, and the amount of Kdo2-lipid A agonist required to reach an equivalent EC50_MAX_ response to that of the control cells was increased by ~12-fold (Figure 6C).

To further support our conclusion that the observed inhibition of IRAK1-dependent TLR signaling was specific, we, as before, assessed TLR3 and TNFR dose responsiveness in our SINV infection model system. As observed above during the ectopic expression of the SINV CP protein, SINV infection did not affect the IRAK1-independent signaling events of TLR3 (Figure 6D). Once again, modest but statistically significant effects were observed in regards to TNFR stimulation during SINV infection.

Collectively, the data presented here show that IRAK1-dependent TLR7 and TLR4 signaling is markedly inhibited during SINV infection, while the IRAK1-independent signaling events of TLR3 and the TNFα receptor were unaffected by SINV infection. These observations largely agree with our previous model system which utilized ectopically expressed CP proteins. However, the magnitude of impact on TLR7 signaling is less striking during infection. Whether this is due strictly to CP expression levels, or an accumulating effect on IRAK1-dependent signaling is unclear at this time. 

### 3.6. SINV Infection Impairs IRAK1-Dependent Signaling during Viral Particle/TLR7 Agonist Co-Exposure

**Figure 7:**
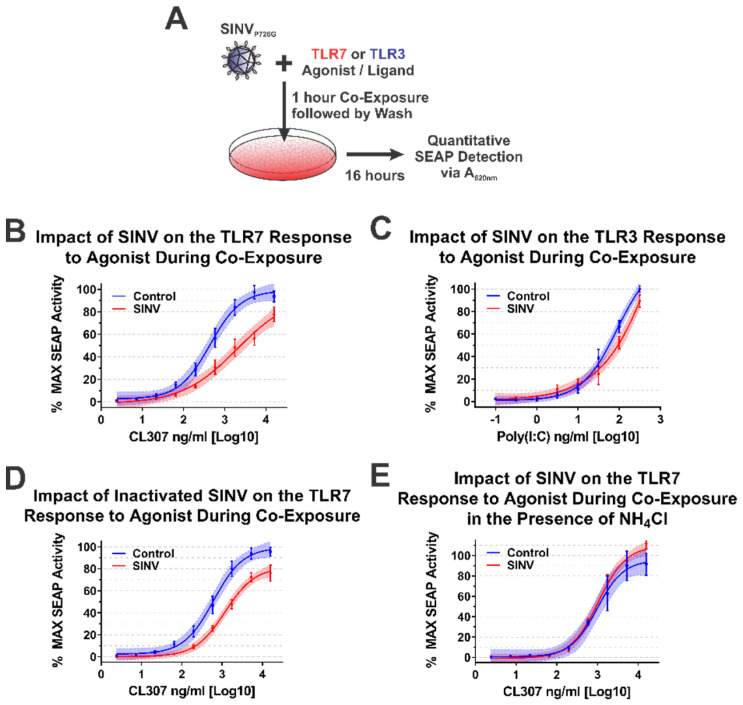
The SINV Capsid Protein Delivered by Incoming Infectious and Non-Infectious Particles Is Sufficient to Inhibit IRAK1-Dependent TLR Signaling. (**A**) A representative diagram of the co-exposure systems used to assess the impact of the incoming SINV CP proteins derived from particles. Specific differences between the experimental designs are noted in the title of each graph. Comparison of the curves in each panel reveals the impact of the CP–IRAK1 interaction on agonist co-exposure during (**B**) delivery of the SINV CP protein from infectious particles in the presence of the TLR7 agonist CL307, (**C**) delivery of the SINV CP protein from infectious particles in the presence of the TLR3 agonist poly(I:C), (**D**) delivery of the SINV CP protein from UV inactivated particles in the presence of the TLR7 agonist CL307, and (**E**) the effect of viral entry inhibitors on the sensing of CL307 by TLR7. In all graphs, mock infected cells are represented by blue lines and data points, and those receiving SINV viral particles are represented by red lines and data points. All quantitative data shown are the minimum of six independent biological replicates conducted over several days with at least two independent SINV preparations. Quantitative data shown are the means of the biological replicates, and the error bars represent the standard deviation of the means. The connecting line represents a non-linear regression of the underlying data, and the shaded region indicates the 95% confidence interval of the non-linear regression. Thus, data points where the shaded regions do not intersect are statistically significant by at least a *p*-value of <0.05, as determined by ANOVA analysis.

During alphaviral infection, there are two stages in which the CP protein may affect host IRAK1-dependent signaling—immediately upon entry to a new host cell when local areas of high CP protein concentrations are formed during nucleocapsid disassembly, or later during infection when the synthesis of new CP protein has commenced [1]. From the data obtained from the ectopic expression studies above, we are able to conclude that the synthesis of CP protein is capable of inhibiting IRAK1-dependent signaling events. Similarly, the quantities of CP protein synthesized during SINV infection are also capable of interfering with IRAK1-dependent TLR signaling. Accordingly, we may reasonably conclude that, in addition to the numerous other changes to the cellular environment, IRAK1-dependent signaling during the later stages of infection is impacted by the SINV CP protein. However, the above data do not indicate whether or not IRAK1-dependent signaling is perturbed by the delivery of SINV CP protein to the cytoplasm of the target cell during viral entry. To test whether the SINV CP protein can negatively impact IRAK1-dependent signaling in an entry model of infection, we utilized a co-exposure system to assess the dose responsiveness of the TLR7 receptor in the presence of SINV particles (Figure 7A). 

As demonstrated by the data in Figure 7B, co-exposure of TLR7 reporter cells with SINV particles and the TLR7 agonist CL307 elicited reduced maximal activation and reduced dose responsiveness by approximately 10-fold relative to control cells which were mock infected during co-exposure. Thus, the SINV CP protein delivered as part of the nucleocapsid core is capable of diminishing the IRAK1-dependent sensing of ssRNA PAMPs during the early stages of infection. It should be noted that the overall reduction in maximal activation was lessened relative to systems with continual CP expression (as in Figure 4, Figure 5 and Figure 6). 

As before, we utilized TLR3 as a means by which the specificity of the inhibition of IRAK1-dependent signaling could be assessed during a co-exposure approach. As shown in Figure 7C, co-exposure of poly(I:C) and infectious SINV particles did not impact the capacity of the cells to sense and respond to TLR3 agonist. These data in conjunction with that described above further secure the conclusion that the SINV CP protein specifically inhibits IRAK1-dependent signaling during infection. 

Careful consideration of the co-exposure approach identifies the possibility that nascent synthesis of the SINV CP protein may be negatively impacting the capacity of the TLR7 reporter cells to respond to agonist exposure. To control for this possibility and assess the specific impact of the incoming viral CP proteins, we redesigned the co-exposure system to utilize UV-inactivated viral particles which are incapable of initiating viral replication, and by extension incapable of de novo expression of the CP protein from the subgenomic RNA. In this system, any effect noted on IRAK1-dependent signaling must be due to components of the incoming viral particles. As shown in Figure 7D, co-exposure of UV-inactivated SINV CP particles and CL307 similarly resulted in decreased TLR7 sensing relative to mock infected co-exposure controls. Hence, the incoming viral CP proteins delivered from non-infectious viral particles are capable of inhibiting IRAK1-dependent signaling. 

Finally, in order to demonstrate that cytoplasmic entry of the SINV CP protein was required for the inhibition of the IRAK1-dependent TLR7 signaling process, we further modified the infectious co-exposure system to include the presence of ammonium chloride, a lysosomotropic salt which prevents the acidification of the endosome during maturation thereby preventing the entry of viral particles [65,66]. Microscopic visualization of the treated cells confirmed the functionality of the ammonium chloride block to viral entry via the lack of GFP expression. Notably, in this system no deficiency in IRAK1-dependent signaling was observed (Figure 7E). Therefore, these data demonstrate that endosomal acidification and the completion of the viral entry pathway leading to the release of the CP protein to the cytoplasm is required for the inhibition of IRAK1-dependent signaling. 

Collectively, these data provide further evidence in support of our initial hypothesis and delineate the impacts of the CP–IRAK1 interaction on IRAK1-dependent signaling during viral entry. Moreover, these data indicate that the fusion of the viral envelope, and presumably the release of the nucleocapsid core into the cytoplasm, is required for the inhibitory effects of the incoming SINV CP protein.

## 4. Discussion

### 4.1. Defining the SINV Protein–Protein Interaction Network

Here we present our efforts using an innovative BioID2 discovery approach to identify novel host–pathogen interactions of the alphaviral CP protein in tissue culture models of infection. Prior to this study, the identification of alphaviral CP protein host–pathogen interactions were limited in scope; and to our knowledge, the unbiased discovery of CP protein–protein interactions was absent from the knowledgebase. The most in depth characterizations of alphaviral CP protein–protein interactions involve those of the VEEV CP protein, which has been shown to interact with elements of the nuclear import/export machinery, and host kinases during infection [64,67,68,69,70,71]. The lack of unbiased discovery efforts in the knowledgebase is likely due to the molecular nature of the alphaviral CP protein, which unfortunately exhibits a high degree of promiscuous binding to commercially available purification resins. The net effect is the substantial precipitation of the alphaviral CP proteins in the absence of target-specific antibodies unless highly stringent binding and wash conditions are used [35]. The harsh wash conditions necessitate the formation of cross-linked complexes prior to purification, as the wash conditions identified through the literature are likely to be incompatible with the purification of native protein–protein interaction complexes. As cross-linking methods form a molecular “snap shot” of the cellular environment, protein–protein interactions which are comparatively rare, temporally regulated, or fleeting in nature are likely to be underrepresented or absent during detection. 

To overcome the challenges associated with alphaviral CP protein–protein interaction discovery, we utilized the BioID2 discovery approach. The covalent addition of a biotin moiety to host factors that come in close proximity to the alphaviral CP proteins enables their subsequent purification under rigorous conditions [36,37]. A key advantage of this approach in that the BioID2 biotin ligase is capable of tagging host protein interactants whose interactions may be exceedingly rare, or those which may be highly transient, as the biotin tag durably remains after the interaction event for subsequent purification. 

As reported by our data above, several host–pathogen interactions were identified via the SINV CP-BioID2 discovery screen. Whether or not these are genuine interactants remains to be determined experimentally; however, we believe these interactions to be bona fide CP–protein interactions for several reasons. First, the lack of extensive intrinsic protein–protein interactions amongst the identified interactants is reflective of the close-proximity requirement of the biotin labeling event during the BioID2 screen. Further evidence of specificity can be obtained from the observations that host proteins with RNA-binding domains, such as RNA-Recognition Motifs (RRMs), KH-type, and Zinc-fingers, are absent from the interactant list, suggesting that the associations of the CP protein with these factors is not simply due to non-specific interactions bridged by an RNA molecule. These observations, coupled with the fact that the BioID2-CP interactants are not biased towards high abundance proteins, provide further evidence that the putative CP–protein interactions are likely to be genuine and functionally meaningful to alphaviral biology.

Review of the putative interactants reveals several of particular interest for future validation and assessment. Amongst these are several host factors involved in the regulation of RNA stability or function, including LARP1, IGF2BP3, TARDBP, STAU1, the N6-methyladenosine readers (m6A) YTHDC2 and YTHDF2, and the Zinc-finger antiviral protein (ZAP)-associated DHX30. It is unclear as to whether the CP interaction serves to deter the interaction of these host factors with the viral genomic RNAs by creating a protective swarm around the incoming genome, or whether they aid in the attraction of beneficial factors to the viral genome via the maintenance of the naCP–RNA interactions after disassembly of the nucleocapsid core. Similarly, it is unknown at this time whether these interactions prevent or disrupt the RNA:Protein and, or protein–protein interactions of the putative interactants. 

Thus, whether these interactions are specifically pro- or antiviral is unknown at this time, and further experimentation is merited. Nonetheless, we are able to provide several predictions/hypotheses based on the defined roles of the aforementioned host factors. LARP1 is known to bind to mRNAs with a 5′ Terminal Oligopyrimidine Motif (5′ TOP) to prevent the association of eIF4E with the 5′ cap structure [72,73,74,75,76]. Therefore, the CP–LARP1 interaction may serve to prevent LARP1 from assembling on the viral RNA to prevent its translation. The interactions of IGF2BP3 with a given mRNA are associated with enhanced RNA stability, therefore this interaction may be an instance where the recruitment of the protein to the viral RNA is beneficial to the viral genome [77,78]. STAU1, or Staufen1, is a component of the Staufen-Mediated Decay (SMD) pathway, which is a highly regulated RNA surveillance pathway which competes with the Nonsense-Mediated Decay (NMD) pathway [79]. As alphaviruses have been previously identified as prime targets for NMD, but are apparently resistant to its effects, the interaction of the CP protein with STAU1 may represent a means by which the NMD pathway is evaded during infection [80]. We hypothesize that the CP–STAU1 interaction, if genuine, may represent a mechanism by which the naCP–RNA interactions serve to stabilize the incoming viral genomic RNAs [35]. The association of TARDBP with an RNA has been reported to attract elements of the cellular deadenylase machinery, specifically Caf1, to enhance the RNA decay in a target specific manner [81]. Consequently, the CP–TARDBP interaction may be another component of the alphaviral RNA’s capacity to resist deadenylation during infection [82,83]. The m6A-associated proteins YTHDF2 and YTHDC2 contribute to the regulation of RNA stability by recruiting the deadenylation machinery, and the 5′→3′ exonuclease XRN1, respectively [84,85,86,87,88,89]. As such, the CP protein may represent a means by which the stability of the incoming viral genomic RNA is further supported. Finally, DHX30 is known to associate and regulate the activity of ZAP [90]. Importantly, ZAP has been previously demonstrated to restrict RNA virus infection, including alphaviral infections [91,92,93,94]. As above, the CP–DHX30 interaction may be a means by which the virus can evade antiviral effectors in the inhospitable cellular environment until later stages of infection when the host cell has been effectively co-opted for viral replication. 

### 4.2. The Host IRAK1 Protein Is a Conserved Interactant of the Alphaviral CP Proteins

Our BiMC experiments demonstrate that the CP–IRAK1 interaction was genuine and not an artefact of the BioID2 discovery approach, and that this particular host–pathogen interaction was conserved across several members of the genus *Alphavirus*. Interestingly, the BiMC data implied that the interaction may be the strongest with the CP proteins of the two encephalitic alphaviruses tested—VEEV and EEEV. While not explicitly tested, the CP protein of Western Equine Encephalitis virus (WEEV) may be reasonably presumed to share the IRAK1 interaction as it is highly similar to that of EEEV. Despite showing significant complementation, the three arthritic alphaviruses tested in this study—SINV, RRV, and CHIKV—showed somewhat reduced BiMC activity relative to encephalitic alphaviruses. The precise implications of this trend are unknown, and further biochemical assessment is warranted prior to concluding that the CP–IRAK1 interaction of the encephalitic CP proteins are indeed superior to those of the arthritogenic viruses. Regardless, these data indicate that the CP–IRAK1 interaction is conserved. 

Comparisons of the alphaviral CP proteins provides few details as to the identity of the necessary and sufficient interaction domains required for the CP–IRAK1 interaction. Broadly speaking the alphaviral CP protein may be subdivided into two domains—a largely disordered positively charged *N*-terminal region, and a *C*-terminal protease domain [95]. The *N*-terminal domain of alphaviruses exhibits considerable sequence divergence outside of unifying characteristic of being highly poly-basic. The *N*-terminal regions of several alphaviruses have been described in more detail, and often regions associated with nucleic acid binding, CP dimerization, and packaging specificity are noted [96,97,98,99,100,101,102,103,104,105]. For several alphaviruses, most notably VEEV, distinct motifs important to the biology of the CP protein have been identified [67,106]. In contrast to the *N*-terminal domain, the *C*-terminal protease domain is largely conserved amongst the members of the genus [95,107]. The data above suggest that the interaction may be mediated by a conserved aspect of the alphaviral CP proteins, which would seemingly implicate the *C*-terminal protease domain. Nonetheless, it is equally likely, perhaps if not more so, that the interaction is mediated by the *N*-terminal domain as this domain is known to facilitate other intermolecular interactions involving the alphaviral capsid proteins. Work designed to delineate the necessary and sufficient CP–IRAK1 interaction determinants are ongoing within the Sokoloski lab. Importantly, such experiments may lead to the creation of interaction deficient viruses, by which the importance of the CP–IRAK1 interaction on viral replication/pathogenesis may be assessed.

### 4.3. The CP–IRAK1 Interaction Negatively Impacts the Detection of TLR Ligands

Taken together, our data demonstrate the importance of the interaction between the alphavirus CP and IRAK1 proteins on IRAK1-dependent signaling in a cellular model of infection. Indeed, from the data shown in Figure 4, Figure 5, Figure 6 and Figure 7, we can reasonably conclude that the SINV CP protein inhibits IRAK1-dependent signaling in a highly specific manner. Nonetheless, the precise mechanism how this occurs is unclear currently. We hypothesize that the CP–IRAK1 interaction serves to disrupt downstream IRAK1 protein–protein interactions or preclude the phosphorylation/activation of IRAK1 [38,39]. 

**Figure 8:**
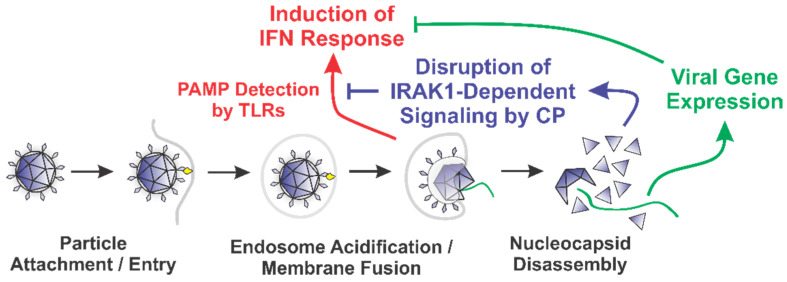
A Diagram of the Roles of the Nucleocapsid Components Early during Infection.

Our data indicate that the SINV CP protein reduces the dose responsiveness of IRAK1-dependent TLRs during ectopic expression and infections of tissue culture models. We posit that the alphaviral CP protein serves to enable the evasion of the host innate immune response by masking the detection of PAMPs via the interruption of the IRAK1-dependent signaling cascade concurrent with the viral entry events and prior to viral gene expression (Figure 8). Importantly, our data indicate that the CP proteins delivered from incoming viral particles, regardless of their infectious potential, were capable of inhibiting TLR7. Therefore, the CP protein is capable of masking PAMP detection in permissive cells, and in non-permissive cells which are exposed to CP protein without viral gene expression. The magnitude of effect is clearly linked to the level of CP protein present in the system, as greater effects were observed in the presence of ongoing CP protein synthesis. Therefore, in addition to be an early mechanism by which the sensing of viral PAMPs by the IRAK1-dependent TLRs may be manipulated, ongoing CP protein expression represents a means by which IRAK1-dependent processes are blunted during the later stages of infection. 

The overarching impact of this phenomenon is the evasion of the direct and collateral activation of an innate immune response without the need for prior intracellular viral gene expression [64,67,108,109,110]. It is likely that this evasion mechanism is highly important to viral replication and dissemination, as alphaviruses are exceptionally sensitive to the effects of type-I IFNs [111,112]. Thus, while alphaviruses have evolved several mechanisms by which the innate immune response may be limited during intracellular replication, the fact that these evasion mechanisms require the accumulation of viral proteins via ongoing viral gene expression creates the necessity of an earlier evasion mechanism to preserve the permissibility of the host environment. We hypothesize that the CP–IRAK1 interaction represents such a mechanism. 

### 4.4. Potential Ramifications of the CP–IRAK1 Interaction Beyond TLR Evasion—The Disruption of IL-1 Signaling

The host IRAK1 kinase is functionally important to cellular signaling events unrelated to the direct sensing of PAMPs. The IRAK1 protein, as can be deduced from its full name—the Interleukin-1 Receptor-Associated Kinase 1—is integrally involved in the sensing of ligands via the IL1R receptor. As the data shown in this study indicate that IRAK1-dependent signaling events are disrupted by the Old World alphaviral CP proteins, we hypothesize that IL1R signaling events may be similarly affected during alphaviral infection, and preliminary efforts confirm that this hypothesis is likely true. 

As IL-1 is a key mediator of the host inflammatory response, interfering with IL1R signaling may have profound impacts on the establishment and resolution of the inflammatory response [113,114,115]. IL-1 has been identified as integral to the formation of arthritis and encephalitis in both infectious and non-infectious settings [116,117]. It should be noted that elevated levels of IL-1 are associated with severe alphaviral disease [118,119,120,121].

During inflammation, the activity/impact of IL-1 is controlled by balancing IL1R signaling through the expression of IL-1, IL-1 responsive genes, and IL1R-antagonists (IL1RAs). As the CP–IRAK1 interaction effectively mutes IRAK1-dependent signaling via an intracellular mechanism, the signals received by the binding of IL-1 to IL1R may not be effectively transduced leading to altered gene expression in cells exposed to CP protein. Importantly, the data shown in this study demonstrate that a permissive infection is not required for the perturbation of IRAK1-dependent signaling, suggesting that bystander cells which are not actively infected may exhibit disrupted signaling profiles. Accordingly, further investigation into the impact of the CP–IRAK1 interaction on IL-1 signaling and host inflammation and pathology is needed.

## 5. Conclusions

Here we have reported the use of an innovative approach to identify the protein–protein interactions of the SINV CP protein. In addition to identifying novel CP–protein interactions, we utilized state-of-the-art model systems to define the interaction of the SINV CP protein with the host IRAK1 protein. Importantly, the CP–IRAK1 interaction negatively impacted the capacity of IRAK1-dependent signaling to occur. While viral entry was required for CP protein-mediated signaling interference, the CP proteins delivered by the incoming viral particles were sufficient to significantly mask TLR7 sensing regardless of their infectious potential. Thus, the CP–IRAK1 interaction masks IRAK1-dependent signaling in permissive and non-permissive cells alike. Taken together, the data presented in this study significantly contribute to the field by (i) establishing the use of a robust discovery approach to identify alphaviral CP–protein interactions, and (ii) delineating a novel mechanism by which the host innate immune system is evaded during the earliest intracellular stages of the alphaviral lifecycle.

## Data Availability

Supporting data regarding the mass spectrometry and ontological.

## Supplementary Materials

The following are available online at https://www.mdpi.com/1999-4915/13/3/377/s1, Supplementary File S1: Mass Spectrometric and Ontological Analyses of the SINV CP BioID2 Screen.
